# Optimization method and experimental research on attitude adjustment scheme of attitude adaptive rescue robot

**DOI:** 10.1038/s41598-022-22991-7

**Published:** 2022-10-26

**Authors:** Shipeng Wang, Dewei Pan, Zhaoxin Zhou, Haochen Yu, Xushi Ma, Guanqing Fang

**Affiliations:** 1 School of Navigation, Shandong Jiaotong University, Weihai, 264200 China; 2 Sino-German Marine Rescue and Equipment Research Center, Shandong Jiaotong University, Weihai, 264200 China; 3 Basic Teaching Department, Shandong Jiaotong University, Weihai, 264200 China; 4 BeiHai Rescue Bureau, Ministry of Transport, Yantai, 264000 China

**Keywords:** Mechanical engineering, Applied mathematics, Computational science, Computer science

## Abstract

To improve the space attitude adjustment efficiency of the robot designed in this study, the average water level height variation of each ballast tank during the rescue process and the ballast water filling mass before the rescue process are taken as optimization variables, the minimal ballasting time during rescue process as the optimisation objective, and the heel and trim inclination angle, and stability in the rescue process as the constraint conditions. For the first time, an optimization method of a rescue robot space attitude adjustment scheme based on a dynamic programming algorithm is proposed. Relevant experiments and data collection were carried out with a model robot with a physical ratio of 1:2. MATLAB simulation and model robot experimental results show that compared with an empirical scheme, the total deployment time and ballast water total allocation mass are reduced by 11.07% and 30.79%, respectively, and the heel and trim angle variation stability is increased by 4.18% and 8.67%, respectively. The optimization model and algorithm are beneficial to improve the space attitude adjustment efficiency and stability of the rescue robot in this paper, and it is also easier to transfer to other fields of ballast water allocation, which has strong practical engineering significance.

## Introduction

In recent years, China’s frequent floods and drowning accidents have caused great losses of life and property to the local governments and people; and have also exposed the lack of professional water rescue equipment^1^. Existing water rescue equipment, whether traditional or intelligent rescue equipment, often only considers surface rescue and “passive” rescue, but does not considered “active” or “salvage” rescue methods for drowning people. Therefore, there is an urgent need to develop a new type of water rescue equipment based on the needs of safe operation and the improvement of rescue efficiency. It is of great significance to meet the requirements of space attitude adjustment speed, control and stability of such rescue equipment.

The status of existing research shows that research on attitude adjustment and optimization of underwater structures is mainly based on ships. It mainly focuses on optimizing ship ballast water allocation, adjusting the damaged ship attitude and calculating the damaged ship stability. In the research part of ship ballast water allocation optimization, we proposed a ship subdivision arrangement method that can ensure the performance of a ship’s sea sequential ballast water exchange (SBWE) in the preliminary design stage. The minimum longitudinal bending moment and shear force a the beam are the goals, and multiple safety criteria of the SBWE are used as constraints. The ballast water allocation optimization model of a gravity-flow composite ballast system and its efficient solution algorithm model improve the construction efficiency of crane ships and reduce energy consumption to provide theoretical and technical support, Chen et al.^2^. A ballast water allocation optimization model and its efficient solution algorithm model of the ballast pump and gravity composite ballast system are established, which provides theoretical and technical support for improving the construction efficiency of crane ships and reducing energy consumption, Ju^3^. Ballasting with water tanks is essential for improving the efficiency, cost and safety of lifting operations with revolving floating cranes. Based on a dynamic programming algorithm, an automatic ballast water system for azimuth cranes is designed, which dynamically allocates the azimuth crane ballast water, realizes the computer dynamic ballast control of large ships, and improves the construction efficiency, Liu et al*.*^4^ proposed a multiobjective evolutionary algorithm (MOEA/D) based on decomposition technology to optimize the ballast water allocation of crane ships. The number of cabins involved in load regulation is reduced by 27%, and the amount of water in regulation is reduced by 24% and 38%, which verifies the feasibility and effectiveness of the MOEA/D algorithm, Zhou et al*.*^5^. In the research part of floating state adjustment when the ship is damaged, the calculation method of the optimal righting measure scheme for damaged ships was studied based on a genetic algorithm (GA) according to the actual damage to the ship. The optimization of righting measures and the comparison with the optimization results of traditional methods proves the advanced nature and engineering practicability of using a GA to optimize the righting measures of damaged ships, Wang et al*.*^6^. To improve the ship’s survivability at sea after damage, based on the SolidWorks three-dimensional software as the research platform, the VB high-level language is used to write the program for secondary development, a visual interface is designed simultaneously, and Microsoft file storage is used to realize the floating adjustment calculation based on the three-dimensional model and real-time simulation in the SolidWorks environment. The corresponding real-time simulation calculation software for floating state adjustment is developed, which has the advantages of visualization and a high degree of automation, Ma^7^. Using a 6-DOF weak nonlinear model to study the possibility of ship surfing, and proposing a ship design stage, the influence of surfing is reduced by calculating and adjusting the centre of buoyancy. This method is a good solution to reduce the influence of waves on the stability of the hull, Yu et al.^8^. In the research part of the ship damage stability calculation, the changes in the spatial position and stress state of the plate during the normal process of the ship after it was damaged and flooded. The hull space mechanics balance equation, hull stability, and plate positive force mathematical model were established, and the straightening process of the damaged hull was simulated using GHS software. The results show that the reasonable arrangement of the alignment point is more conducive to the alignment of the capsizing hull, Pan et al.^9^. Damage stability under medium-wave conditions experimental results show that the damage of the bow compartment has the most serious influence on the stability, Yang et al.^10^. The phenomenon of a “rapid capsize” during the intermediate stages of flooding may represent a potentially dangerous situation for a ship. A mathematical model of the ship’s damaged cabin is established, and the influence of the ship’s instantaneous water stability is verified experimentally. The experimental results show that the subdivision method has a stronger application value than the traditional method, Vermeer et al*.*^11^.

In summary, domestic and foreign scholars have not studied the space attitude adjustment of wading rescue robots, which is still a gap in the engineering field. This article is the first, to propose an optimization method for a rescue robot space attitude adjustment scheme based on a dynamic programming algorithm proposed. The preballast allocation quality before the rescue operation and the average water level change of each ballast tank during the allocation are taken as the optimization variables, the minimum ballast time and the total ballast water allocation quality in the lifting rescue process are taken as the optimization objectives, and an optimization model of efficient ballast water allocation of the robot is established. This optimization model based on dynamic programming algorithm divides the multi-stage ballast water deployment decision problem into several small stages at the “lift” height interval of the rescue operation for the whole rescue process, which simplifies the complexity of the calculation, and is more suitable for the working characteristics of this rescue robot. It solves the problems of efficient allocation and optimization of ballast water and adjustment and maintenance of space attitude for the rescue robot. It lays a theoretical foundation for the development and application of a computerized ballast water automatic ballast system for rescue robots. It can also be applied to other engineering fields such as special engineering ships or underwater robots that need to adjust the spatial attitude, such as special crane ships, rotary crane ships, diving ships, underwater rescue robots, underwater dredging robots and underwater exploration robots. Secondly, the thinking mode and modeling method of this paper can also have a certain theoretical reference significance for the research on attitude adjustment and control in aerospace fields such as quadrotor UAV, space exploration satellite and launch vehicle. It is more conducive to quickly rescuing a drowning person and adapting to its underwater posture, and improving the success rate of rescue, which has strong practical engineering significance.

## Rescue robot operation mode and ballast system

**Figure 1:**
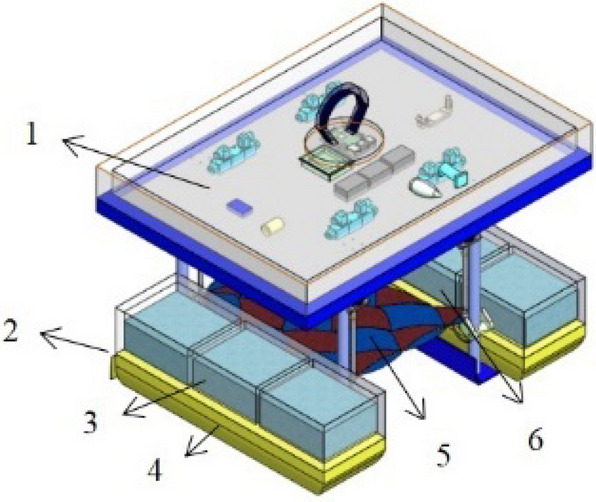
Conceptual model of the rescue robot.

**Figure 2:**
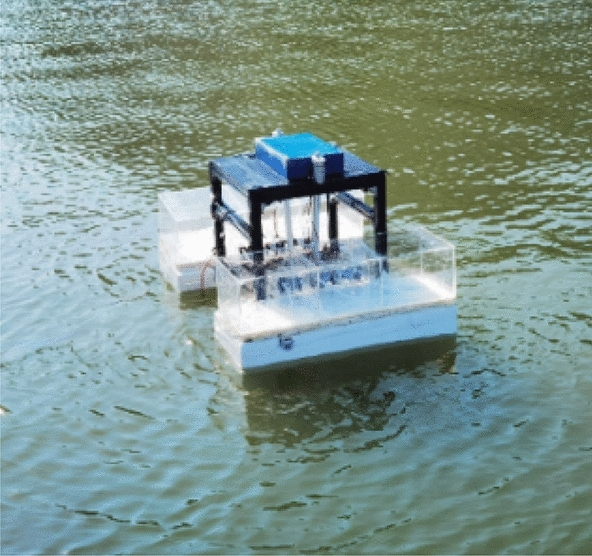
Model machine.

Based on the structure and operating characteristics of the rescue robot, a conceptual model diagram and model machine experiment diagram are shown in Figs. 1 and 2 below. When rescuing a drowning person, it is necessary to reasonably select, determine and implement a ballast water allocation scheme according to the actual posture of the drowning person to adapt to the underwater posture of the drowning person, take the initiative or assist in “salvage” rescue, and improve the rescue efficiency and safety. This chapter introduces the rescue methods and ballast water allocation methods of the robot, which lays the foundation for the subsequent optimization modelling and model robot experiments.

The parts of the structure shown in Fig. 1 are as follows: 1. control box; 2. propeller and steering gear; 3. rescue robot water tank and water pump; 4. polyethylene floating body; 5. rescue net; and 6. hydraulic telescopic rod.

### Rescue method

The rescue robot designed and manufactured in this study is different from existing water rescue tools. It can form a ballast water allocation system through the design of the ballast water tank, to change the space posture in the water and adapt to a drowning person, which is beneficial to improve the rescue efficiency and safety.

In the rescue and salvage lifting process, a drowning person is different from the general cargo transportation. During the actual rescue, due to the different weights of drowning persons, there is a high centre of gravity and large span. Therefore, it is necessary to carry out ballast water repeated deployment to offset the horizontal, longitude and vertical moments caused by the lifting of the drowning person. If ballast water is not allocated, it will not meet the requirements of the safety of rescue operations.

**Figure 3:**
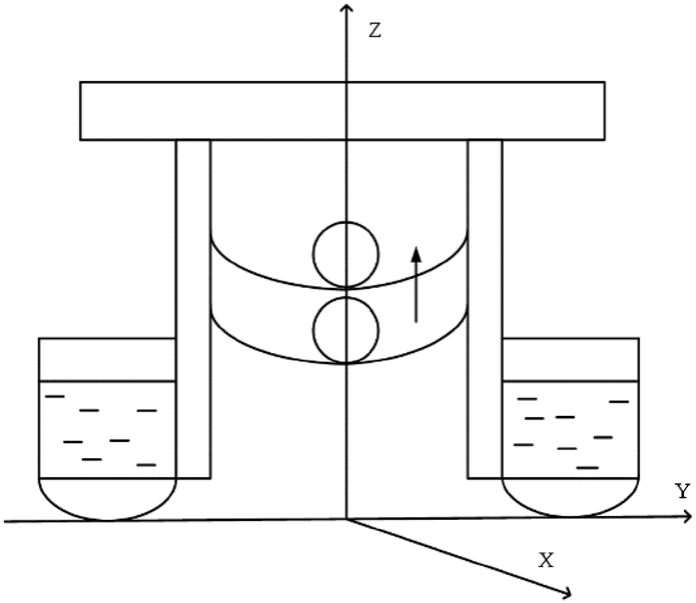
Robot rescue method.

The Cartesian coordinate system is taken as the prototype to establish the space coordinate system of the rescue robot. The intersection of the datum plane, cross section and longitudinal section of the rescue robot is regarded as the coordinate origin, and the bow, port and vertical directions of the robot define the X, Y and Z respectively, as shown in Fig. 3. The circle in Fig. 3 represents the drowning person.

### Ballast method

**Figure 4:**
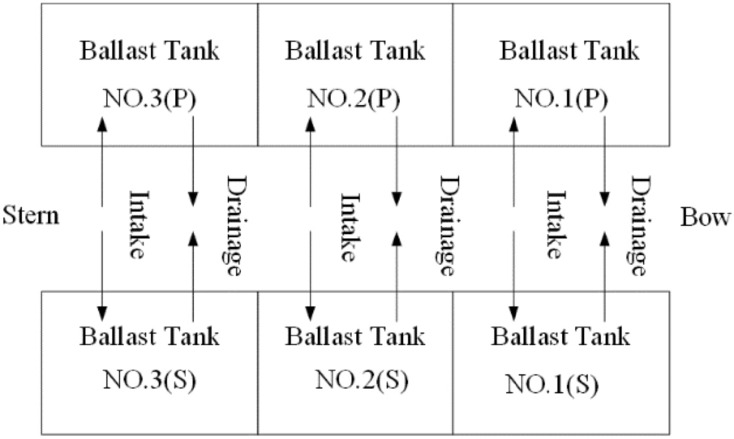
Ballast tank and ballast pump planning and layout.

Generally, in the design and manufacture of small and medium-sized water vehicles, a self-suction ballast pump is widely used because of its simple structure, durability, reliability and high efficiency, which is more in line with the actual application of rescue robots^12,13^. Therefore, a self-suction ballast pump is selected to build the robot’s ballast water ballast system, and 6 ballast water tanks are used. The capacity of the water tank and the displacement of the ballast pump meet the basic experimental requirements. The layout of the ballast water tanks is shown in Fig. 4.

## External disturbance and constraint factors

As shown above, the deployment of ballast water not only has a great impact on the efficiency, stability and cost of salvage and salvage operations, but also determines the safety of salvage operations.

This chapter analyses the external and internal influencing factors generated when the rescue robot ballast water, such as the influence of the free liquid surface, moment and wind drift on the initial stability, and safety considerations, such as the gyration speed, heel and trim angle, ballast tank water level and height are constrained. This lays the foundation for the establishment of an efficient allocation model of rescue robot ballast water based on a dynamic programming algorithm.

### External disturbance factors

On the premise of meeting the basic stability balance requirements, the rescue robot needs to break the balance condition to change the spatial attitude in the water. The existence of an external disturbance on the free surface, wind, current and wave moment in the ballast tank and the existence of safety constraints will also affect the stability of the robot and attitude.

#### Initial stability calculation of the rescue robot under the influence of the ballast water free surface

In the rescue process, considering the stability of a small inclination angle (inclination angle less than 15°), the robot will be affected by different moments. Ballast water should be allocated as much as possible to adjust the attitude and stability, which should meet the following mathematical equation:

1 $$\tau_{{\text{w}}} + \tau_{{\text{r}}} + \tau_{{\text{p}}} + \tau_{{\text{h}}} = \left( {\tau_{{{\text{wx}}}} + \tau_{{{\text{wy}}}} } \right) + \tau_{{\text{r}}} + \left( {\tau_{{{\text{px}}}} + \tau_{{{\text{py}}}} } \right) + \tau_{{\text{h}}} ,$$

where $\tau_{{\text{w}}}$, $\tau_{{\text{r}}}$, $\tau_{{\text{p}}}$ and $\tau_{{\text{h}}}$ represent the moment of the ballast water, the tilt moment of the rescued person, the restoring moment of the robot and the disturbance moment of the external environment, respectively; $\tau_{{{\text{wx}}}}$, $\tau_{{{\text{wy}}}}$, $\tau_{{{\text{px}}}}$ and $\tau_{{{\text{py}}}}$ represent the component moments of the ballast water and rescued person in the *X*- and *Y*-axis directions, respectively.

The lateral and pitch moments are:

2 $$\tau_{{\text{w}}} = \tau_{{{\text{wx}}}} + \tau_{{{\text{wy}}}} = \sum\limits_{i = 1}^{{\text{n}}} {\rho \Delta d_{i}^{p,s} } m_{i} \Delta x_{i}^{p,s} + \sum\limits_{i = 1}^{{\text{n}}} {\rho \Delta d_{i}^{p,s} } m_{i} \Delta y_{i}^{p,s} ,$$

where *n* represents the number of ballast tanks; $\rho$ represents the density of water; $\Delta d_{i}^{p,s}$ represents the average height change of the ballast water in the ith ballast tank on the port and starboard sides; and $\Delta x_{i}^{p,s}$ and $\Delta y_{i}^{p,s}$ are the moving distances of the centre of gravity of ballast water in the i-th ballast tank on the port and starboard sides in the *X*- and *Y*-axis directions, respectively.

The tilting moment $\tau_{{\text{p}}}$ of the rescued person can be expressed as:

3 $$\tau_{{\text{p}}} = \tau_{{{\text{px}}}} + \tau_{{{\text{py}}}} = m_{p} g\Delta x_{p} + m_{p} g\Delta y_{p} + m_{p} g\Delta z_{p} ,$$

where $\Delta x_{p}$, $\Delta y_{p}$ and $\Delta z_{p}$ represent the moving distances of the rescued person’s centre of gravity in the X, Y and Z directions, respectively; $m_{p}$ represents the mass of the rescued person; and *g* is gravitational acceleration.

**Figure 5:**
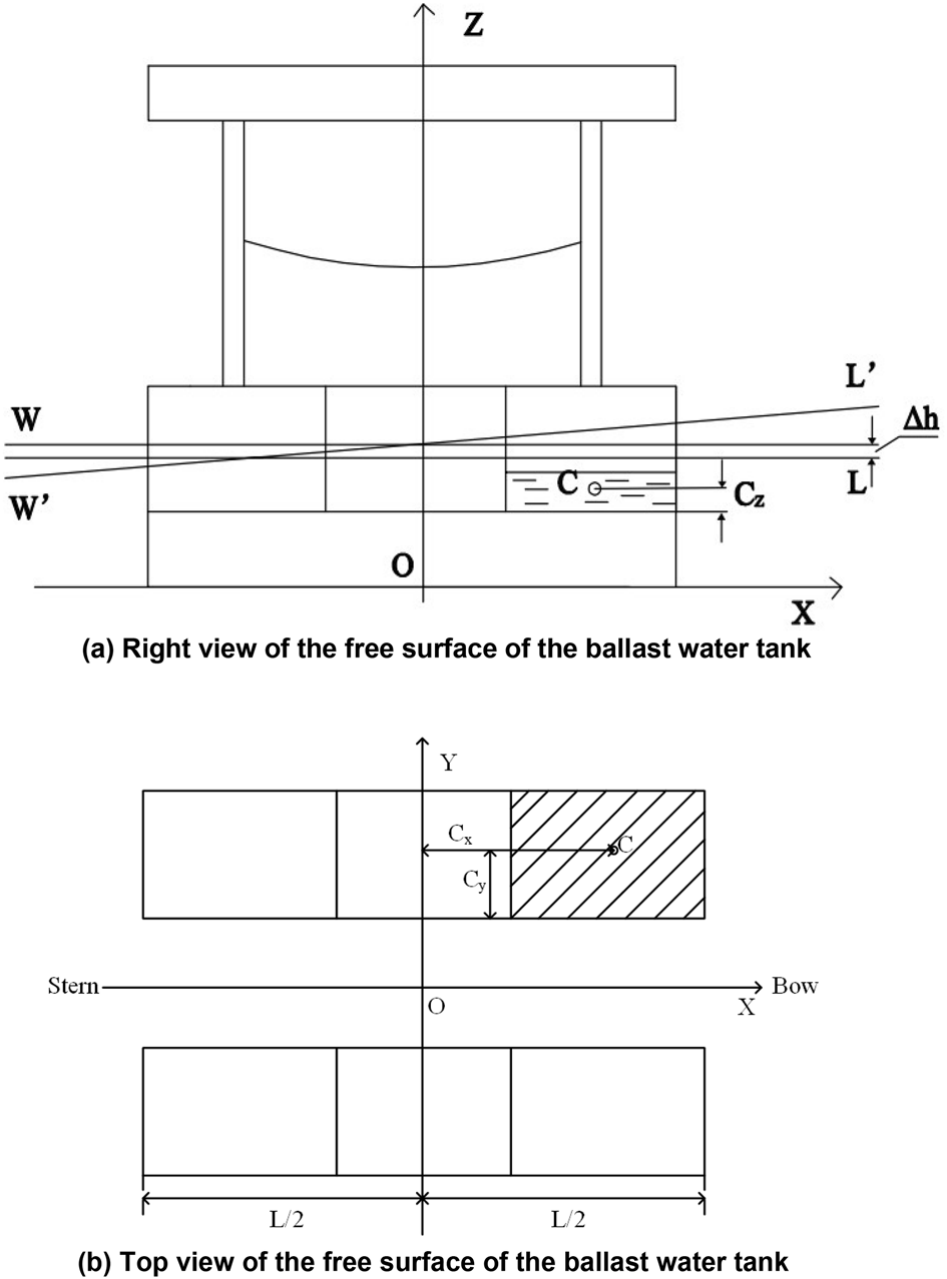
Robotic ballast tank under a free surface.

Suppose the robot initially floats at waterline *W*_0_*L*_0_. After lifting and rescuing the drowning person, it floats at waterline *W*_1_*L*_1_. The water in the ballast tank tilts and floats at waterline *W*_2_*L*_2_. The incremental average draught at this moment is denoted by Δ*h*. The stability analysis diagram of the robot under the influence of the free surface is shown in Fig. 5. Figure 5a shows the right view of the free surface ballast water tank, Fig. 5b shows the top view of the free surface ballast water tank, and *C* indicates the centroid of the water in the ballast water tank.

The original barycentric coordinates *A*_*g*_ of the robot are (*x*_*g*_, *y*_*g*_, *z*_*g*_), and the initial barycentric coordinates of the rescued person are *A*_*p*_(*x*_*p*_, *y*_*p*_. *z*_*p*_) and move to the barycentric coordinates *Aʹ*_*p*_(*xʹ*_*p*_, *yʹ*_*p*_, *zʹ*_*p*_), the centre coordinates of the loaded ballast water are *A*_*w*_(*x*_*w*_, *y*_*w*_, *z*_*w*_), that is, before the process of lifting the rescued person, the height of the robot’s transverse and longitudinal stability are expressed as:

4 $$\begin{aligned} \overline{{G_{1} M}}_{NT} = & \overline{GM} \\ & + \frac{{m_{p} g + m_{W} g}}{{D + m_{p} g + m_{W} g}}\left[ {h + \frac{\Delta h}{2} - \left( {z_{p} + z_{w} } \right)\overline{GM} } \right] \\ \overline{{G_{1} M}}_{NL} = & \overline{GM}_{L} \\ & + \frac{{m_{p} g + m_{W} g}}{{D + m_{p} g + m_{W} g}}\left[ {h + \frac{\Delta h}{2} - \left( {z_{p} + z_{w} } \right)\overline{GM}_{L} } \right]. \\ \end{aligned}$$

The heel angles $\varphi$ and trim angles $\theta$ are expressed as:

5 $$\begin{array}{*{20}l} \varphi = \arctan \frac{{m_{p} gy_{p} + m_{W} gy_{w} }}{{\left( {D + m_{p} g + m_{W} g} \right)\overline{{G_{1} M}}_{NT} }}, \hfill \\ \theta = \arctan \frac{{m_{p} g\left( {{\text{x}}_{p} - x_{f} } \right) + m_{w} g\left( {x_{w} - x_{f} } \right)}}{{\left( {D + m_{p} g + m_{W} g} \right)\overline{{G_{1} M}}_{NL} }}, \hfill \\ \end{array}$$

where $\overline{{G_{1} M}}_{NT}$ and $\overline{{G_{1} M}}_{NL}$ represent the new transverse and longitudinal stability height after the rescue personnel, respectively; $x_{f}$ represents the height of the drift centre on the water plane; *G* is the centre of gravity of the robot; *D* is the drainage volume of the robot; *M* is the stable centre of the robot; *L* is the shape length; *B* is the profile width; $\varphi$ is the heel angle; $\theta$ is the trim angle; $m_{W}$ represents the total mass of the loaded ballast water; and $x_{w}$ and $y_{w}$ represent the transverse and transverse centre of gravity coordinates of the ballast water, respectively.

Then the restoring moment $\tau_{{\text{r}}}$ of the robot is:

6 $$\tau_{{\text{r}}} = \left( {D + m_{p} g + m_{w} g} \right)\overline{{G_{1} M}}_{NT} \sin \varphi .$$

#### Analysis and calculation of wind, current and wave moment under external environment disturbance

The moment $\tau_{{\text{h}}}$ caused by the external environment is as follows:

7 $$\tau_{{\text{h}}} { = }\tau_{wind} { + }\tau_{wave} { + }\tau_{flow} ,$$

where $\tau_{wind}$, $\tau_{wave}$ and $\tau_{flow}$ represent the wind moment, wave moment and flow moment, respectively.

The modular method divides different parts above the structural waterline into different modules for separate calculation. Clearly, the modular method is more suitable for calculating the wind torque of rescue robots, and the modular method is often suitable for offshore engineering and other small and medium-sized aquatic structures. The calculation steps and formulas are as follows^14,15^:

8 $$V_{{H_{L} }} = V_{{H_{Lr} }} \left( {\frac{{H_{L} }}{{H_{Lr} }}} \right)^{\Lambda } ,$$

where $V_{{H_{L} }}$ is the wind speed when the height of the horizontal plane is $H_{L}$; $H_{Lr}$ is the reference height, which is 10 m; $V_{{H_{Lr} }}$ is the wind speed at the reference height; and ${\Lambda }$ is a coefficient, generally 0.1–0.15.

Then, the average wind speed of the superstructure above the entire water surface is:

9 $$V_{{\text{a}}}^{2} = \frac{1}{A}\iint {V^{2} }\left( {y,z} \right)dydz,$$

where *A* is the sum of the total wind-receiving area and $V^{2} \left( {y,z} \right)$ is the wind speed at the coordinates $\left( {y,z} \right)$ of the wind-receiving surface.

Then in different components, the phoenix moment of the first module is:

10 $$\tau_{{wind_{\xi } }} = \frac{1}{2}\rho_{air} T_{s\xi } T_{h\xi } V_{\xi a}^{2} A_{\xi } ,$$

where $\rho_{air}$ represents the air density; $T_{s\xi }$ and $T_{h\xi }$ represent the shape and height factors of the $\xi$th module, respectively; and $A_{\xi }$ represents the wind-receiving area of the $\xi$th module.

Then, the total wind moment is:

11 $$\tau_{wind} = \sum\limits_{\xi = 1}^{{S_{L} }} {\tau_{{wind_{\xi } }} } ,$$

where $\tau_{wind}$ represents the total wind load and $S_{L}$ represents the total number of divided modules.

Using a slice theory calculation method based on Faltinsen, the convection load is divided into transverse and longitudinal calculations^16,17^.

1. *Estimation of the transverse flow moment* The transverse flow moment experienced by the robot can be calculated by estimating the method in still water as:
  12 $$\tau_{{flow_{x} }} = \frac{1}{2}\rho V_{a}^{2} T_{{flow_{x} }} S_{w} \cos \psi \left| {\cos \alpha } \right|,$$
  where $\tau_{{flow_{x} }}$ denotes the resistance coefficient; *Re* denotes the Reynolds number; $L_{s}$ denotes the total length of the robot; $S_{w}$ denotes the robot wet surface area; $\alpha$ represents the flow angle; and ${\uppsi }$ represents the yaw angle.
  In this case, the resistance coefficient $\tau_{{flow_{x} }} = T_{1} (1+T)T_{f}$; the model conversion coefficient ${\text{T}}_{{1}} { = }\frac{{5}}{{\left| {\cos {\upalpha }} \right|}}$; the friction resistance coefficient of the plate $T_{{\text{f}}} = \frac{0.075}{{\left( {\lg {\text{Re}} - 2} \right)^{2} }}$; and the Reynolds number ${\text{Re = }}\frac{{{\text{V}}_{{\text{c}}} {\text{L}}_{{\text{s}}} }}{{\text{v}}}$.
2. *Estimation of the longitudinal flow moment* The longitudinal flow moment experienced by the robot can be estimated using the principle of longitudinal flow as:
  13 $$\tau_{{flow_{{\text{y}}} }} =\, \frac{1}{2}\rho \left[ {\int_{L} {dxT_{D} \left( x \right)d\left( x \right)} } \right]V_{a}^{2} \sin \left( \alpha \right)\left| {\sin \left( \alpha \right)} \right|,$$
  where *T* denotes the shape factor; $T_{D} \left( x \right)$ is the drag coefficient of the robot cross-section at ordinate x; and $d\left( x \right)$ is the draught height at the cross section.

Therefore, the total flow torque experienced by the robot is

14 $$\begin{aligned} \tau_{flow} = & \frac{1}{2}\rho V_{a}^{2} T_{{flow_{x} }} S_{w} \cos \psi \left| {\cos \alpha } \right| \\ & + \frac{1}{2}\rho \left[ {\int_{L} {dxT_{D} \left( x \right)d\left( x \right)} } \right]V_{a}^{2} \sin \left( \alpha \right)\left| {\sin \left( \alpha \right)} \right|. \\ \end{aligned}$$

When calculating the regular wave disturbance force, the wave moment $\tau_{wave}$ is described by the Froude–Krylov assumption, as follows^18^:

15 $$\tau_{wave} = - \iiint_{\tau } {\frac{\partial \Delta P}{{\partial y}}}xd\tau ,$$

where $\tau$ is the inflow volume of the ship and $\Delta P$ is the dynamic pressure caused by waves.

Then, the pressure gradient is^19^:

16 $$\begin{gathered} \frac{\partial \Delta P}{{\partial x}} = \rho gake^{ - kz} \cos \chi \sin \left( {kx\cos \chi - ky\sin \chi - \omega_{e} t} \right), \hfill \\ \frac{\partial \Delta P}{{\partial y}} = - \rho gake^{ - kz} \sin \chi \sin \left( {kx\cos \chi - ky\sin \chi - \omega_{e} t} \right), \hfill \\ \end{gathered}$$

where *a* and *k* are parameters, denoted as $k = \frac{2\pi }{\lambda }$, $a = \rho g\left( {1 - e^{ - kd} } \right)/k^{2}$, *λ* is the wavelength, *d* is the draught height; *χ* is the wave direction angle, also known as the encounter angle, which is used to describe the angle of the wave based on the tail line and takes the counter clockwise direction as positive, ranging from 0 to 2π; $\omega_{e}$ represents the encounter frequency, $\omega_{e} { = }\omega - V\cos \chi$, $\omega$ represents the wave received frequency, *V* is the speed; *x*, *y* and *z* are the horizontal, longitude and vertical coordinates, respectively; and *t* is the encounter time.

The wave moment is^20,21^:

17 $$\begin{array}{*{20}l} \tau_{wave} = \rho gak\sin \chi \int_{L} {\frac{{\sin \left( {k\frac{B\left( x \right)}{2}\sin \chi } \right)}}{{k\frac{B\left( x \right)}{2}}}} e^{ - kd\left( x \right)} \hfill \\ A\left( x \right)\sin \left( {kx\cos \chi - \omega_{e} t} \right)xdx. \hfill \\ \end{array}$$

Then, in the rescue process, the initial barycentric coordinates *A*_*p*_(*x*_*p*_, *y*_*p*_, *z*_*p*_) of the rescued person move to the barycentric coordinates *A’*_*p*_ (*x’*_*p*_, *y’*_*p*_, *z’*_*p*_); then, the heel angles $\varphi$ and trim angles $\theta$ are:

18 $$\begin{gathered} \varphi = \arctan \frac{{m_{p} g\left( {y_{p}^{{\prime}} - y_{p} } \right) + m_{W} gy_{w} }}{{\left( {D + m_{p} g + m_{W} g} \right)\overline{{G_{1} M}}_{NT} }}, \hfill \\ \theta = \arctan \frac{{m_{p} g\left( {{\text{x}}_{p}^{{\prime}} - x_{p} } \right) + m_{w} gx_{w} }}{{\left( {D + m_{p} g + m_{W} g} \right)\overline{{G_{1} M}}_{NL} }}. \hfill \\ \end{gathered}$$

#### Summary of the initial stability calculation of the rescue robot when it is disturbed by the outside world

**Figure 6:**
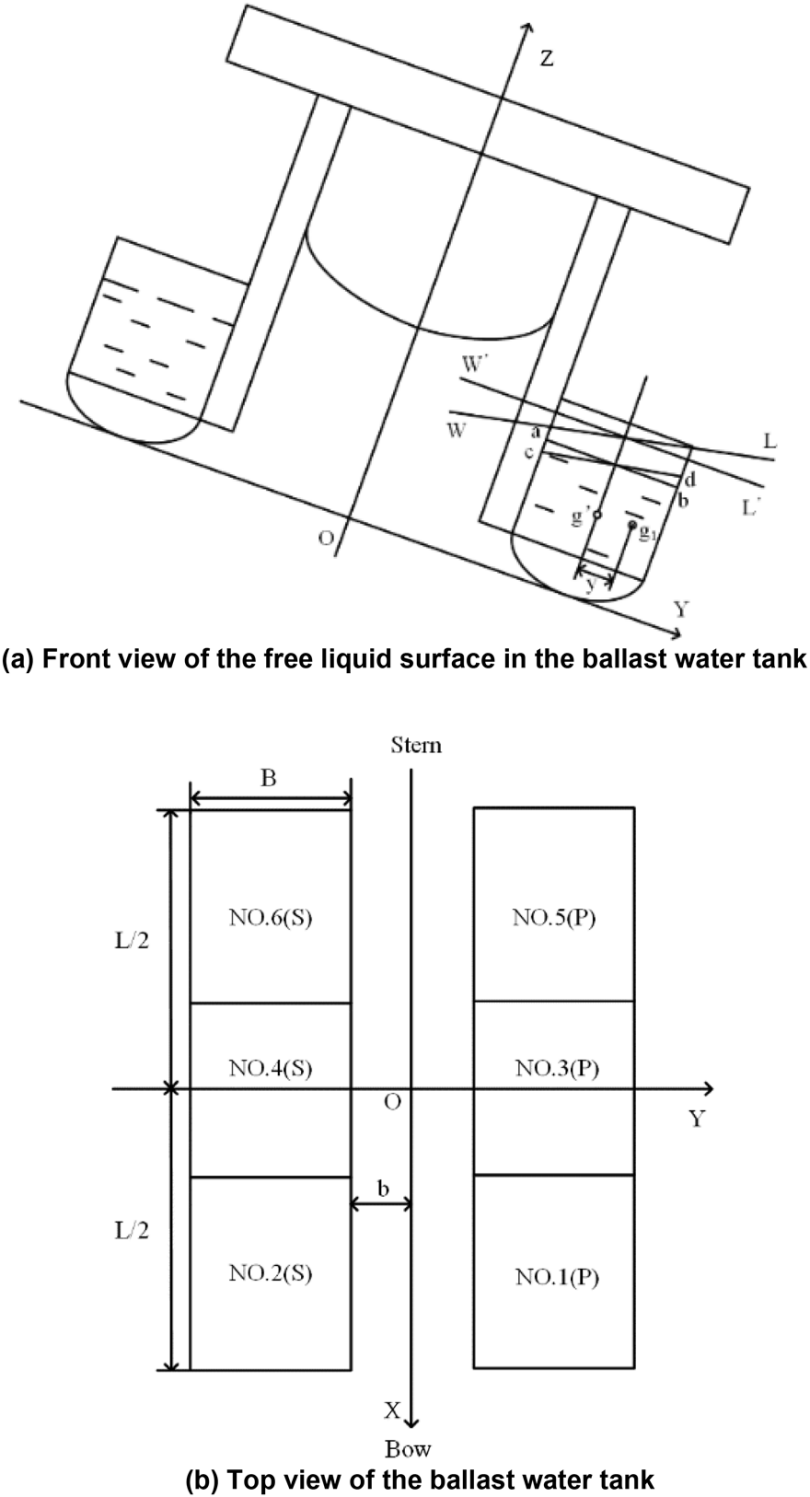
Influence of the free liquid surface stability in the ballast tanks.

The free surface *ad* in the ballast water tank is parallel to the waterline *W*′*L*′, and its centre of gravity is at point *g*′. When the robot heels a small angle *φ*, the free surface of the liquid in the ballast tank is also inclined to become *cd*, it is parallel to the new waterline *WL*, and its centre of gravity moves from point *g*′ to point *g*_1_. Figure 6a shows the front view of the ballast water tank with a free surface, and Fig. 6b shows the top view of the ballast water tank.

When the liquid in the tank is transferred from one side to the other due to the tilt angle, an additional tilt moment $\tau_{wh}$ is generated as follows:

19 $$\tau_{wh} = \rho i_{x} \sin \varphi ,$$

where $i_{x}$ represents the lateral moment of inertia of the free surface area relative to its inclined axis.

Let the overall length of the robot be *L*, the width be *B*, and the centre distance be *b*. Then, in heeling, the moment of inertia of the free liquid to its inclined axis is:

20 $$\begin{aligned} i_{x} &= \frac{1}{12}\frac{L}{3}B^{3} , \hfill \\ i_{Y} &= \frac{1}{12}B\left[ \frac{L}{3} \right]^{3} . \hfill \\ \end{aligned}$$

Then, the actual restoring torque corrected according to the free surface is:

21 $$\tau_{nr} = \left( {D + m_{p} g + m_{W} g} \right)\left[ {\overline{{G_{1} M}}_{NT} - \frac{{\rho i_{x} }}{{\left( {D + m_{p} g + m_{W} g} \right)}}} \right]\sin \varphi ,$$

where $- \frac{{\rho i_{x} }}{{\left( {D + m_{p} g + m_{W} g} \right)}}$ is the correction value of the out-of-stability height of the free liquid surface, which is only related to the size and displacement of the free liquid surface and is unrelated to the volume of the free liquid surface.

Then for the transverse stability heights $\overline{{G_{1} M}}_{NT}$ and longitudinal stability heights $\overline{{G_{1} M}}_{NL}$

22 $$\begin{gathered} \overline{{G_{1} M}}_{NT} = \overline{GM} - 6\frac{{\rho i_{x} }}{{\left( {D + m_{p} g + m_{W} g} \right)}}, \hfill \\ \overline{{G_{1} M}}_{NL} = \overline{GM}_{L} - 6\frac{{\rho i_{{\text{y}}} }}{{\left( {D + m_{p} g + m_{W} g} \right)}}. \hfill \\ \end{gathered}$$

In summary, the final transverse $\overline{{G_{1} M}}_{NT}^{{\prime}}$ and longitudinal stability height $\overline{{G_{1} M}}_{NL}^{{\prime}}$ of the robot are:

23 $$\begin{aligned} \overline{{G_{1} M}}_{NT}^{{\prime}} = & \overline{GM} \\ & + \frac{{m_{p} g + m_{W} g}}{{D + m_{p} g + m_{W} g}}\left[ {h + \frac{\Delta h}{2} - \left( {z_{p} + z_{w} } \right)\overline{GM} } \right] \\ & - \;6\frac{{\rho i_{x} }}{{\left( {D + m_{p} g + m_{W} g} \right)}} ,\\ \overline{{G_{1} M}}_{NL}^{{\prime}} = & \overline{GM}_{L} - \frac{{m_{p} g + m_{W} g}}{{D + m_{p} g + m_{W} g}}\overline{GM}_{L} \\ & - \;6\frac{{\rho i_{y} }}{{\left( {D + m_{p} g + m_{W} g} \right)}}. \\ \end{aligned}$$

### Constraint factors

To describe the parameters and safety of the robot, this section sets some constraints to prevent the robot from overturning and other dangerous situations.

1. Maintain heel angle and trim angle of the robot
  24 $$\begin{gathered} 0^\circ \le \varphi = \arctan \frac{{m_{p} gy_{p} + m_{w} gy_{w} }}{{\left( {D + m_{p} g + m_{w} g} \right)\overline{{G_{1} M}}_{NT} }} \le 15^\circ , \hfill \\ 0^\circ \le \theta = \arctan \frac{{m_{p} g\left( {x_{p} - x_{f} } \right) + m_{w} g\left( {x_{w} - x_{f} } \right)}}{{\left( {D + m_{p} g + m_{w} g} \right)\overline{{G_{1} M}}_{NL} }} \le 15^\circ. \hfill \\ \end{gathered}$$
2. The water level of the ballast water tank is lower than a maximum limit:
  25 $$0 \le d_{i}^{P,S} \le d_{iH}^{P,S} ,$$
  where $d_{i}^{P,S}$ represents the water level height of the ith water tank on the port and starboard sides and $d_{iH}^{P,S}$ represents the maximum height limit of the ith water tank on the port and starboard sides.
3. The amount of preloaded ballast water should not exceed the maximum amount of limited water:
  26 $$0 \le Q_{1}^{P,S} \le Q_{1\max }^{P,S} ,$$
  where $Q_{1\max }^{P,S}$ represents the maximum limited ballast amount of the preballast.
4. *Lifting height limit* The height of lifting the drowning person is limited and cannot exceed a maximum lifting height $H_{{{\text{max}}}}^{K}$ of the robot, that is,
  27 $$H_{{{\text{mix}}}}^{K} \le H^{K} \le H_{{{\text{max}}}}^{K} ,$$
  where $H_{{{\text{mix}}}}^{K}$ and $H_{{{\text{max}}}}^{K}$ represent the minimum and maximum lift heights, respectively, and $H^{K}$ represents the lift height at the K-stage.
5. *Weight limits for rescued drowning persons* When the robot performs the lifting operation of the rescue task, the weight of the rescued person is limited and cannot exceed the maximum load-bearing limit of the robot $P_{\max }^{K}$; that is, $P_{\max }^{K}$ means that the maximum weight of the person to be carried without ballast is:
  28 $$0 \le P^{K} \le P_{\max }^{K} ,$$
  where $P_{\max }^{K}$ represents the maximum weight of the rescued person under the condition of no ballast and $P^{K}$ represents the weight of the rescued person.
6. *Draught limit* The maximum draught limits of the robot in the upright float, heel, trim, and any inclination are $d_{\max }$, $d_{T\max }$, $d_{L\max }$ and $d_{S\max }$, respectively, and the range is:
  29 $$\begin{aligned} d_{{{\text{mi}} x}} \le & d^{K} \le d_{\max } \\ d_{{T{\text{mi}} x}} \le & d_{T}^{K} = \frac{{{\text{d}}_{p}^{K} + {\text{d}}_{S}^{K} }}{2} \le d_{T\max } \\ d_{{L{\text{mi}} x}} \le & d_{L}^{K} = \frac{{{\text{d}}_{F}^{K} + {\text{d}}_{S}^{K} }}{2} \\ & + \frac{{{\text{d}}_{p}^{K} - {\text{d}}_{S}^{K} }}{{L_{S} }} \le d_{L\max } \\ d_{{S{\text{mi}} x}} \le & d_{S}^{K} = \frac{{{\text{d}}_{FP}^{K} + {\text{d}}_{FS}^{K} + {\text{d}}_{SP}^{K} + {\text{d}}_{SS}^{K} + {\text{d}}_{p}^{K} + {\text{d}}_{S}^{K} }}{6} \\ & + \frac{{{\text{d}}_{p}^{K} - {\text{d}}_{S}^{K} }}{{L_{S} }} \le d_{S\max } , \\ \end{aligned}$$
  where $d^{K}$, $d_{T}^{K}$, $d_{L}^{K}$ and $d_{S}^{K}$ represent the average draught of the robot in the state of upright float, heel, trim or any inclination at stage *K*, respectively; $d_{{{\text{mi}} x}}$, $d_{{T{\text{mi}} x}}$, $d_{{L{\text{mi}} x}}$ and $d_{{S{\text{mi}} x}}$ represent the robot in upright float and heel, respectively , the maximum limit of draught in trim or any inclination; ${\text{d}}_{FP}^{K}$, ${\text{d}}_{FS}^{K}$, ${\text{d}}_{SP}^{K}$ and ${\text{d}}_{SS}^{K}$ represent the port and starboard draughts on six sides, respectively.
7. *Rotation speed, angle, and radius limits* The speed, angle and radius of the robot’s rotation are limited as follows:
  30 $$\begin{gathered} 0 \le s^{K} \le s_{\max }^{K} \hfill \\ 0 \le \theta^{K} \le \theta_{\max }^{K} \hfill \\ 0 \le {\text{r}}^{K} \le r_{\max }^{K} , \hfill \\ \end{gathered}$$
  where $s_{\max }^{K}$, $\theta_{\max }^{K}$ and $r_{\max }^{K}$ represent the robot’s maximum gyration speed, angle and radius, respectively.

## Dynamic programming algorithm model

**Figure 7:**
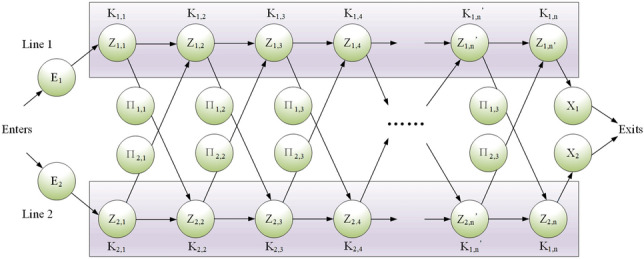
Dynamic programming multistage decision concept diagram.

The ballast water allocation process of rescue robots is a multistage decision-making problem, which is a continuous dynamic process. The advantage of dynamic programming algorithms is that they can be combined with related engineering problems to divide a continuous process into multiple interrelated stages. During the solution process, the optimization results of the previous stage are continuously used to carry out the optimization solution of the next stage. The ballast water allocation problem of rescue robots is regarded as a continuous problem, and a dynamic programming algorithm is used to solve the optimal decision^22–26^. The conceptual diagram of the dynamic programming algorithm is shown in Fig. 7.

Figure 7 clearly shows the advantages and characteristics of the algorithm in the face of multistage multiattribute decision-making problems, that is, in stage *K*_n,m_ under different states *Z*_n,m_, by selecting different decision-making strategies *Π*_n,m_ in the stage, the next optimal stage *K*_n+i,m+j_, and the global optimal solution are reach.

In summary, this research mainly studies the process of ballast water allocation. By actively “salvaging” drowning persons or assisting them to climb a rescue network, a lifting operation is carried out. During the rescue process, a certain lifting height interval is used as a stage variable to make real-time dynamic decisions and destroy or stabilize the posture or stability of the robot in the water, reduce the water regulation and distribution, improve the ballast water allocation efficiency, minimize the water tank inlet/discharge time as the optimization goal, and take the stage space, state space, decision space and state transition equation to establish a mathematical model for the optimal solution of the inlet/discharge of ballast water tank based on a dynamic programming algorithm.

### Stage space

The stage space is usually a finite, infinite or continuous set of points. The finite stage space can be defined as the set of *K* as:

31 $$K = \left( {1,2, \ldots ,N} \right).$$

The infinite set of stage spaces is:

32 $$K = \left( {1,2, \ldots } \right).$$

### State space

The state refers to the description of the system after it makes a decision at a certain stage. This study defines all the states of the system as *Z*, which is called the state space. Any state ${\text{Z}}_{{\upkappa }}$ at any stage *N* belongs to *Z*, denoted as ${\text{z}}_{{\text{k}}} \in {\text{Z}}$. Therefore, the state space can also be expressed as:

33 $$Z = \left( {Z_{1} ,Z_{2} , \ldots ,ZK, \ldots ,Z_{N} } \right),K = 1,2, \ldots ,N.$$

The decision-making stage set *K* of the process is divided into *N* + 2 stages based on the lifting height interval of each segment. For convenience of calculation, the ballast water tanks on both sides are regarded as standard cuboids. The port and starboard sides are represented by the letters *P* and *S*, respectively, and the water level height of each ballast tank on both sides at different decision-making stages is taken as the state set. Then, the state space is represented as $Z_{K}^{P}$ and $Z_{K}^{S}$, respectively, which is collectively called $Z_{K}^{P,S}$, that is:

34 $$Z_{K}^{P,S} = \left( {d_{K1}^{P,S} ,d_{K2}^{P,S} , \ldots ,d_{Ki}^{P,S} , \ldots ,d_{Kn}^{P,S} } \right),\;i = 1,2, \ldots ,n;\;K = 1,2, \ldots ,N + 2,$$

where ${{d}}_{{{{Ki}}}}^{{{P,S}}}$ represents the average water level in the ith ballast tank on the port and starboard sides of the robot under the *N*th stage, and ${{d}}_{{{{Ki}}}}^{{2{P,S}}}$ can be divided into $d_{{K{\text{i}}}}^{P}$ and $d_{{K{\text{i}}}}^{S}$.

### Action space

The action space is usually a random decision-making choice that describes a certain state from a certain stage *K* to the next stage *K* + 1^27,28^. According to the actual robot operating principle, the water level change trend of each ballast tank at different decision-making stages on both sides can be defined as the uncertainty action space *D*:

35 $$D_{Ki}^{P,S} = \left( {ri,lr,st} \right),\;i = 1,2, \ldots ,n;\;K = 1,2, \ldots ,N + 2,$$

where $D_{ki}^{P,S}$ represents the random action set of the *i*th ballast tank on the port or starboard side of the robot in the *N*th stage; *ri* corresponds to the increase in the water level; *lr* corresponds to the decrease in the water level; and *st* corresponds to the level of the water level.

### Decision space

When making a random action decision, it is necessary to express the quantified action effect in mathematical form. Therefore, the action sets of the port and starboard are defined as $D_{K}^{P} \left( {Z_{K}^{P} ,Z_{K}^{P} } \right)$ and $D_{K}^{S} \left( {Z_{K}^{S} ,Z_{K}^{S} } \right)$, respectively, which can be collectively referred to as $D_{K}^{P,S} \left( {Z_{K}^{P,S} ,\Pi_{K}^{P,S} } \right)$, that is:

36 $$D_{K}^{P,S} \left( {Z_{K}^{P,S} ,\Pi_{K}^{P,S} } \right) = \left[ {D_{K1}^{P,S} \left( {Z_{K1}^{P,S} ,\Pi_{K1}^{P,S} } \right),D_{K2}^{P,S} \left( {Z_{K2}^{P,S} ,\Pi_{K2}^{P,S} } \right), \ldots ,D_{Ki}^{P,S} \left( {Z_{Ki}^{P,S} ,\Pi_{Ki}^{P,S} } \right), \ldots ,D_{Kn}^{P,S} \left( {Z_{Kn}^{P,S} ,\Pi_{Kn}^{P,S} } \right)} \right],\;i = 1,2, \ldots ,n;\;K = 1,2, \ldots ,N + 2,$$

where $D_{K}^{P,S} \left( {Z_{K}^{P,S} ,\Pi_{K}^{P,S} } \right)$ represents the decision-making effect of the *i*-th ballast tank on the port and starboard sides in the *N*-th stage under action space *D*; $\Pi_{K}^{P,S}$ represents the change in the average water level, which is called the strategy space.

The port and starboard sides are denoted as $\Pi_{K}^{P}$ and $\Pi_{K}^{S}$, respectively, collectively referred to as $\Pi_{K}^{P,S}$, that is:

37 $$\Pi_{K}^{P,S} = \left( {\Delta d_{K1}^{P,S} ,\Delta d_{K2}^{P,S} , \ldots ,\Delta d_{Ki}^{P,S} , \ldots ,\Delta d_{Kn}^{P,S} } \right),\;i = 1,2, \ldots ,n;\;K = 1,2, \ldots ,N + 2,$$

where $\Delta d_{Ki}^{P,S}$ can be divided into $\Delta d_{Ki}^{P}$ and $\Delta d_{Ki}^{S}$, where $\Delta d_{Ki}^{P}$ represents the average water level height change of the *i*th ballast tank under the *N*th stage on the port side, and $\Delta d_{Ki}^{S}$ represents the average water-level height change of the *i*th ballast tank under the *N*th stage on the starboard side.

### State transition function

A state transition function is constructed to represent the functional process expression when state $Z_{K}$ reaches the next state $Z_{K + 1}$ under the influence of an action $D_{K}$ and a decision space $\Pi_{K}^{P,S}$. The transition state $Z_{K + 1}$ can be obtained according to the state transition function *F*, which can be expressed as $F_{K}^{P} \left[ {Z_{K}^{P} ,D_{K}^{P} \left( {Z_{K}^{P} ,\Pi_{K}^{P} } \right)} \right]$ and $F_{K}^{S} \left[ {Z_{K}^{S} ,D_{K}^{S} \left( {Z_{K}^{S} ,\Pi_{K}^{S} } \right)} \right]$, collectively referred to as $F_{K}^{P,S} \left[ {Z_{K}^{P,S} ,D_{K}^{P,S} \left( {Z_{K}^{P,S} ,\Pi_{K}^{P,S} } \right)} \right]$, that is:

38 $$\begin{aligned} Z_{K + 1}^{P,S} &= F_{K}^{P,S} \left[ {Z_{K}^{P,S} ,D_{K}^{P,S} \left( {Z_{K}^{P,S} ,\Pi_{K}^{P,S} } \right)} \right] \hfill \\ &= Z_{K}^{P,S} + D_{K}^{P,S} \left( {Z_{K}^{P,S} ,\Pi_{K}^{P,S} } \right) \hfill \\ i &= 1,2, \ldots ,n;K = 1,2, \ldots ,N + 2 \hfill \\ \end{aligned}$$

where $F_{K}^{P,S} \left[ {Z_{K}^{P,S} ,D_{K}^{P,S} \left( {Z_{K}^{P,S} ,\Pi_{K}^{P,S} } \right)} \right]$ is the general expression of the state transition function of the port and starboard *N*-th stage. Among them, $Z_{1}^{P} = d_{0}^{P}$ and $Z_{1}^{S} = d_{0}^{S}$.

### Expectation function

The expectation function is the quantitative function used to measure and evaluate the advantages and disadvantages of the selected strategy.

In this study, $S_{K}^{P,S} \left[ {Z_{K}^{P,S} ,D_{K}^{P,S} \left( {Z_{K}^{P,S} ,\Pi_{K}^{P,S} } \right)} \right]$ is used to represent the index when the decision made in state $Z_{K}^{P,S}$ is $D_{K}^{P,S} \left( {Z_{K}^{P,S} ,\Pi_{K}^{P,S} } \right)$ in the *N*th stage; that is, the ballast time in the *N*th stage is:

39 $$S_{1}^{P,S} \left[ {Z_{1}^{P,S} ,D_{1}^{P,S} \left( {Z_{1}^{P,S} ,\Pi_{1}^{P,S} } \right)} \right] = \max \left\{ {\frac{{\rho m_{i}^{P,S} \left( {d_{1i}^{P,S} - d_{0i}^{P,S} } \right)}}{{q_{s}^{P,S} }}} \right\},\;i = 1,2, \ldots ,n;K = 1,$$

40 $$S_{K}^{P,S} \left[ {Z_{K}^{P,S} ,D_{K}^{P,S} \left( {Z_{K}^{P,S} ,\Pi_{K}^{P,S} } \right)} \right] = \max \left\{ {\frac{{\rho m_{i}^{P,S} \Delta d_{Ki}^{P,S} }}{{q_{s}^{P,S} }}} \right\},\;i = 1,2, \ldots ,n;K = 1,2, \ldots ,N + 1,$$

41 $$S_{N + 2}^{P,S} \left[ {Z_{N + 2}^{P,S} ,D_{N + 2}^{P,S} \left( {Z_{N + 2}^{P,S} ,\Pi_{N + 2}^{P,S} } \right)} \right] = \max \left\{ {\frac{{\rho m_{i}^{P,S} \left( {d_{{\left( {N + 1} \right)i}}^{P,S} - d_{{\left( {N + 2} \right)i}}^{P,S} } \right)}}{{q_{s}^{P,S} }}} \right\},\;i = 1,2, \ldots ,n;K = N + 2,$$

where $S_{1}^{P,S} \left[ {Z_{1}^{P,S} ,D_{1}^{P,S} \left( {Z_{1}^{P,S} ,\Pi_{1}^{P,S} } \right)} \right]$ represents the total expected value under a certain action decision in the first stage of the port and starboard during the attitude preadjustment process before rescue; $S_{K}^{P,S} \left[ {Z_{K}^{P,S} ,D_{K}^{P,S} \left( {Z_{K}^{P,S} ,\Pi_{K}^{P,S} } \right)} \right]$ represents the total expected value under the action decision from the second stage to the *N* + 1th stage of the spatial attitude adjustment in the rescue process; $S_{N + 2}^{P,S} \left[ {Z_{N + 2}^{P,S} ,D_{N + 2}^{P,S} \left( {Z_{N + 2}^{P,S} ,\Pi_{N + 2}^{P,S} } \right)} \right]$ represents the total expected value under the *N* + 2th stage action decision in the post salvage drainage process; $p$ is the density of ballast water; and $m_{i}^{P,S}$ represents the bottom area of the ith ballast tank on the port and starboard sides.

### State-action Q-value function

In summary, the Bellman equation expression of the state-action *Q*-value function of all stages can be derived according to the above calculation process and expressed as a recursive description as follows:

42 $$\begin{aligned} Q_{1}^{P,S} \left[ {Z_{1}^{P,S} ,D_{1}^{P,S} \left( {Z_{1}^{P,S} ,{\Pi }_{1}^{P,S} } \right)} \right] = & S_{1}^{P} \left[ {Z_{1}^{P} ,D_{1}^{P} \left( {Z_{1}^{P} ,{\Pi }_{1}^{P} } \right)} \right] \\ & + S_{1}^{S} \left[ {Z_{1}^{S} ,D_{1}^{S} \left( {Z_{1}^{S} ,{\Pi }_{1}^{S} } \right)} \right], \\ \end{aligned}$$

43 $$\begin{aligned} Q^{P,S} \left[ {Z,D\left( {Z,{\Pi }} \right)} \right] = & S_{1}^{P,S} + S_{N + 2}^{P,S} \\ & + \sum\limits_{K = 2}^{N + 1} {\left\{ {S_{K}^{P,S} \left[ {Z_{K}^{P,S} ,D_{K}^{P,S} \left( {Z_{K}^{P,S} ,{\Pi }_{K}^{P,S} } \right)} \right]} \right\}} . \\ \end{aligned}$$

The optimal Q-value function is defined as $Q_{K}^{P,S}$ and expressed as the expected sum of stage *N*:

44 $$\begin{gathered} Q_{K}^{P,S} \left[ {Z_{K}^{P,S} ,D_{K}^{P,S} \left( {Z_{K}^{P,S} ,{\Pi }_{K}^{P,S} } \right)} \right] = \min \left\{ {\sum\limits_{i = 1}^{K} {S_{K}^{P,S} \left[ {Z_{K}^{P,S} ,D_{K}^{P,S} \left( {Z_{K}^{P,S} ,{\Pi }_{K}^{P,S} } \right)} \right]} } \right\}. \hfill \\ \hfill \\ \end{gathered}$$

45 $$Q_{K}^{P,S} \left[ {Z_{K}^{P,S} ,D_{K}^{P,S} \left( {Z_{K}^{P,S} ,\Pi_{K}^{P,S} } \right)} \right] = \min \left\{ {S_{K}^{P,S} \left[ {Z_{K}^{P,S} ,D_{K}^{P,S} \left( {Z_{K}^{P,S} ,\Pi_{K}^{P,S} } \right)} \right] + Q_{K - 1}^{P,S} \left[ {Z_{K - 1}^{P,S} ,D_{K - 1}^{P,S} \left( {Z_{K - 1}^{P,S} ,\Pi_{K - 1}^{P,S} } \right)} \right]} \right\}.$$

Then, the optimal state-action *Q*-value function Bellman equation expression is expressed as:

46 $$Q^{ * P,S} \left[ {Z,D\left( {Z,{\Pi }} \right)} \right] = \mathop {\min }\limits_{{D\left( {Z,{\Pi }} \right)}} Q_{K}^{P,S} \left[ {Z_{K}^{P,S} ,D_{K}^{P,S} \left( {Z_{K}^{P,S} ,{\Pi }_{K}^{P,S} } \right)} \right].$$

The maximum expected value is obtained when the optimal *Q*-value function is determined. Thus, the action strategy is also optimal, which satisfies:

47 $$D^{ * } \left( {Z,{\Pi }} \right) \in \arg \mathop {\min }\limits_{{\Pi }} Q^{ * P,S} \left[ {Z,D\left( {Z,{\Pi }} \right)} \right].$$

This action policy, $D^{ * } \left( {Z,{\Pi }} \right)$, is said to be greedy for the *Q*-value function. Therefore, if the optimal $Q^{ * P,S} \left[ {Z,D\left( {Z,{\Pi }} \right)} \right]$ value can be determined, the greedy action can be calculated, and the optimal action strategy $D^{ * } \left( {Z,{\Pi }} \right)$ can be obtained.

Therefore, according to the derivation of the above expression, the optimal action strategies of the port and starboard sides can be easily obtained, which can be recursively expressed as:

48 $$\begin{array}{*{20}l} Q_{1}^{ * P,S} \left[ {Z_{1}^{P,S} ,D_{1}^{P,S} \left( {Z_{1}^{P,S} ,{\Pi }_{1}^{P,S} } \right)} \right] = \min \left\{ {S_{1}^{P,S} \left[ {Z_{1}^{P,S} ,D_{1}^{P,S} \left( {Z_{1}^{P,S} ,{\Pi }_{1}^{P,S} } \right)} \right]} \right\}, \hfill \\ Q_{2}^{ * P,S} \left[ {Z_{2}^{P,S} ,D_{2}^{P,S} \left( {Z_{2}^{P,S} ,{\Pi }_{2}^{P,S} } \right)} \right] = \min \left\{ {S_{2}^{P,S} \left[ {Z_{2}^{P,S} ,D_{2}^{P,S} \left( {Z_{2}^{P,S} ,{\Pi }_{2}^{P,S} } \right)} \right]} \right\}. \hfill \\ \cdots \hfill \\ \end{array}$$

Considering the first two stages of the port side as an example, the optimal action strategy of the port side:

49 $$\begin{gathered} D_{1}^{ * P} \left( {Z_{1}^{P} ,{\Pi }_{1}^{P} } \right) = \left\{ {D_{11}^{ * P} \left( {Z_{11}^{P} ,\Delta d_{11}^{ * P} } \right),D_{11}^{ * P} \left( {Z_{11}^{P} ,\Delta d_{12}^{ * P} } \right), \ldots ,D_{1i}^{ * P} \left( {Z_{1i}^{P} ,\Delta d_{1i}^{ * P} } \right), \ldots ,D_{1n}^{ * P} \left( {Z_{1n}^{P} ,\Delta d_{1n}^{ * P} } \right)} \right\}, \hfill \\ D_{2}^{ * P} \left( {Z_{2}^{P} ,{\Pi }_{2}^{P} } \right) = \left\{ {D_{21}^{ * P} \left( {Z_{21}^{P} ,\Delta d_{21}^{ * P} } \right),D_{21}^{ * P} \left( {Z_{21}^{P} ,\Delta d_{22}^{ * P} } \right), \ldots ,D_{2i}^{ * P} \left( {Z_{2i}^{P} ,\Delta d_{2i}^{ * P} } \right), \ldots ,D_{2n}^{ * P} \left( {Z_{2n}^{P} ,\Delta d_{2n}^{ * P} } \right)} \right\}. \hfill \\ \end{gathered}$$

Thus, the optimal action strategy of each stage is obtained recursively according to the optimal strategy of the port side is:

50 $$\begin{gathered} D_{1}^{ * P} \left( {\Pi_{1}^{ * P} } \right) = \left\{ {\Delta d_{11}^{ * P} ,\Delta d_{12}^{ * P} , \ldots ,\Delta d_{1i}^{ * P} , \ldots ,\Delta d_{1n}^{ * P} } \right\}, \hfill \\ D_{2}^{ * P} \left( {\Pi_{2}^{ * P} } \right) = \left\{ {\Delta d_{21}^{ * P} ,\Delta d_{22}^{ * P} , \ldots ,\Delta d_{2i}^{ * P} , \ldots ,\Delta d_{2n}^{ * P} } \right\}. \hfill \\ \end{gathered}$$

That is, the optimal policy set under the port optimal action is:

51 $${\text{h}}_{{1\left( {N + 2} \right)}}^{ * P} = D_{1}^{ * P} \left( {{\Pi }_{1}^{ * P} } \right),D_{2}^{ * P} \left( {{\Pi }_{2}^{ * P} } \right), \ldots ,D_{K}^{ * P} \left( {{\Pi }_{K}^{ * P} } \right), \ldots ,D_{N + 2}^{ * P} \left( {{\Pi }_{N + 2}^{ * P} } \right).$$

The optimal deployment plan for the port side is then obtained. Similarly, the optimal strategy set for the starboard side is derived as follows:

52 $${\text{h}}_{{1\left( {N + 2} \right)}}^{ * S} = \left\{ {D_{1}^{ * S} \left( {{\Pi }_{1}^{ * P} } \right),D_{2}^{ * S} \left( {{\Pi }_{2}^{ * S} } \right), \ldots ,D_{K}^{ * S} \left( {{\Pi }_{K}^{ * S} } \right), \ldots ,D_{N + 2}^{ * S} \left( {{\Pi }_{N + 2}^{ * S} } \right)} \right\}.$$

Then, the optimal strategy set of the overall system can be easily obtained as:

53 $$h_{{1\left( {N + 2} \right)}}^{ * P,S} = \left\{ {D_{1}^{ * P,S} \left( {\Pi_{1}^{ * P,S} } \right),D_{2}^{ * P,S} \left( {\Pi_{2}^{ * P,S} } \right), \ldots ,D_{K}^{ * P,S} \left( {\Pi_{K}^{ * P,S} } \right), \ldots ,D_{N + 2}^{ * P,S} \left( {\Pi_{N + 2}^{ * P,S} } \right)} \right\}.$$

Finally, a calculation flow chart of the optimal allocation scheme of ballast water for attitude-adaptive rescue robots based on a dynamic programming algorithm can be obtained, as shown in Fig. 11.

## Experimental analysis

The rescue of drowning persons is a special type of rescue and rescue mission. Whether it is a rescue in still water, a flood, the sea, or other environments, there are high requirements for the professional quality of rescuers and the safety and reliability of the rescue equipment. It has the characteristics of high technical requirements, high risk and unpredictability.

According to the relevant research report of the Fire Rescue Detachment of Ningbo City, Zhejiang Province, China, when a drowning person is in distress, the instinctive reaction is to struggle upright, flutter up and down, or two postures of “standing upright” and “floating upside down”, and they will try their best to grasp everything that can be done. Shao proposed that three basic situations may occur when a drowning person is drowning^29^:

1. The drowning person is still conscious, and there is still struggle and a need for help.
2. The drowned person is confused and has no signs of struggle.
3. The drowning person is still in the water. They completely disappear and sink into the water.

**Figure 8:**
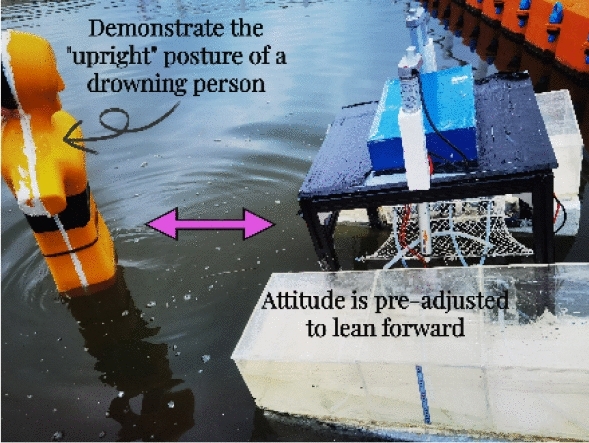
Experimental procedure (a).

**Figure 9:**
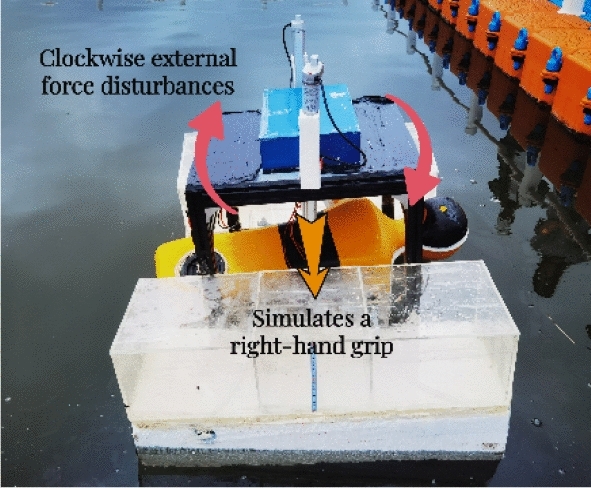
Experimental procedure (b).

**Figure 10:**
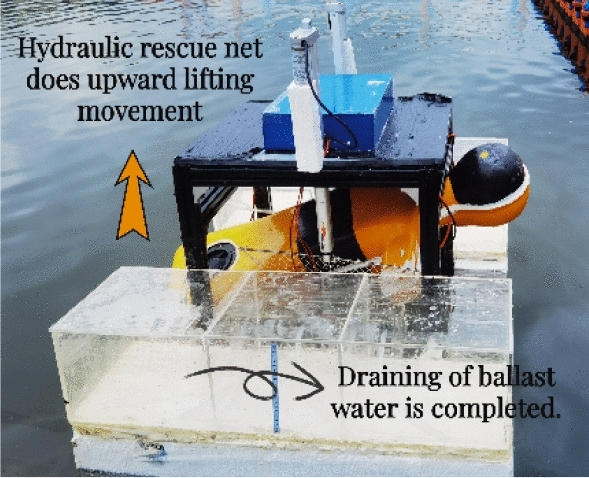
Experimental procedure (c).

Combined with existing research literature, accident cases and the functional and structural characteristics of the rescue robot, the experimental background set in this paper is mainly aimed at the simulated rescue scene for the drowning person, taking the simulated rescue scene in a conscious and “upright” posture as an example, as shown in Figs. 8, 9 and 10.

- First, when people fall into the water, they are conscious, the robot posture is adjusted to adapt to the person falling into the water, and facilitate them to climb the rescue net.
- During the climbing process, first the right hand contacts the rescue net, and then the left hand continues to climb until the body lies on the rescue net. The influence of the person falling into the water on the stability and posture of the robot is simulated through an artificial intervention experiment.
- Finally, the lifting movement is performed through a hydraulic rescue net, and the ballast water is emptied to complete the operation.

Generally, industry experts who have been engaged in water rescue and salvage for many years can judge and choose a rescue plan under the background of any rescue environment through their rich practical experience and fast and simple calculation. Therefore, an expert from the North Sea Rescue Bureau of the Ministry of Transport is invited to conduct on-site ballast water allocation experiments on the rescue robot under the above experimental background to obtain empirical allocation data. MATLAB data simulation and physical model experiment analysis are carried out. The scale parameters of the experimental model machine are shown in Table 1, the experimental environment parameters are shown in Table 2, and the cabin size of the model ballast tank under the layout of Fig. 4 is shown in Table 3. There is a preballast process before the salvage lifting movement. After the operation, all ballast water is emptied at once time to complete a rescue operation.

**Table 1:** Model robot parameters.

Project	Parameter
Total length/cm	87 cm
Width/cm	96 cm
High/cm	80 cm
Maximum lifting capacity/kg	50 kg
Maximum lift height/cm	40 cm
Ballast pump flow	2L/min

**Table 2:** Parameters of the marine environment loads.

Wind scale	Wind direction	Wave scale	Wave direction
2–3	90°	2	90°

**Table 3:** Parameters of the ballast tanks.

Project	Total length/cm	Mould breadth/cm	Bottom area/cm^2^	Volume/cm^3^	Capacity/L
NO1 (P)	31	30	900	18,000	18
NO2 (S)	31	30	900	18,000	18
NO3 (P)	25	30	750	15,000	15
NO4 (S)	25	30	750	15,000	15
NO5 (P)	31	30	900	18,000	18
NO6 (S)	31	30	900	18,000	18

**Figure 11:**
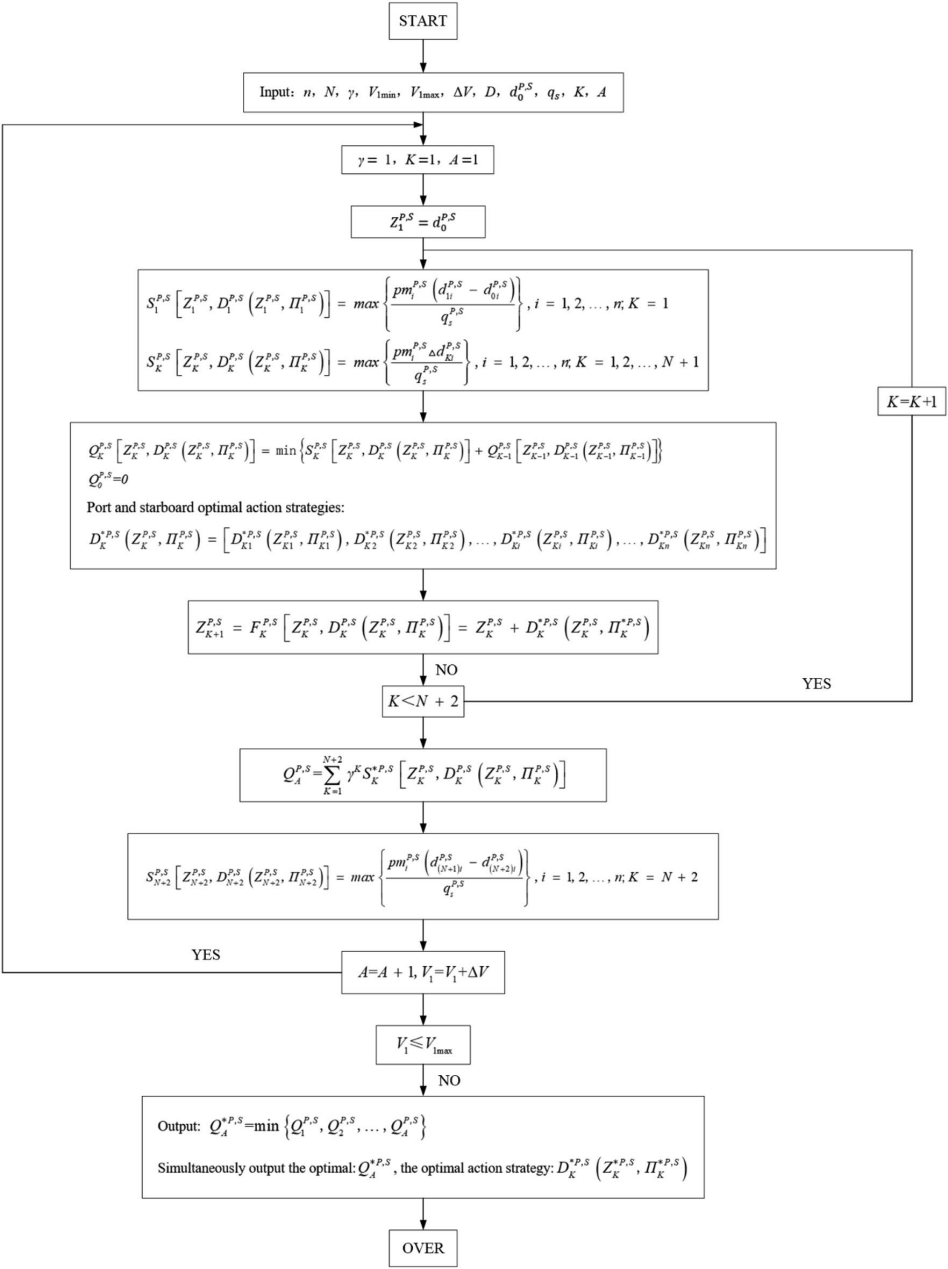
Calculation flow chart of the optimization mode.

The whole rescue process is limited by the maximum lifting height of 40 cm, and divided into 40 stages with an interval of 1 cm. Each 1 cm lifting is a stage to solve step by step. According to the empirical data, Fig. 11 shows the calculation steps to solve the optimized ballast scheme.

### Comparison of the average water level data changes between experience and optimization schemes

**Figure 12:**
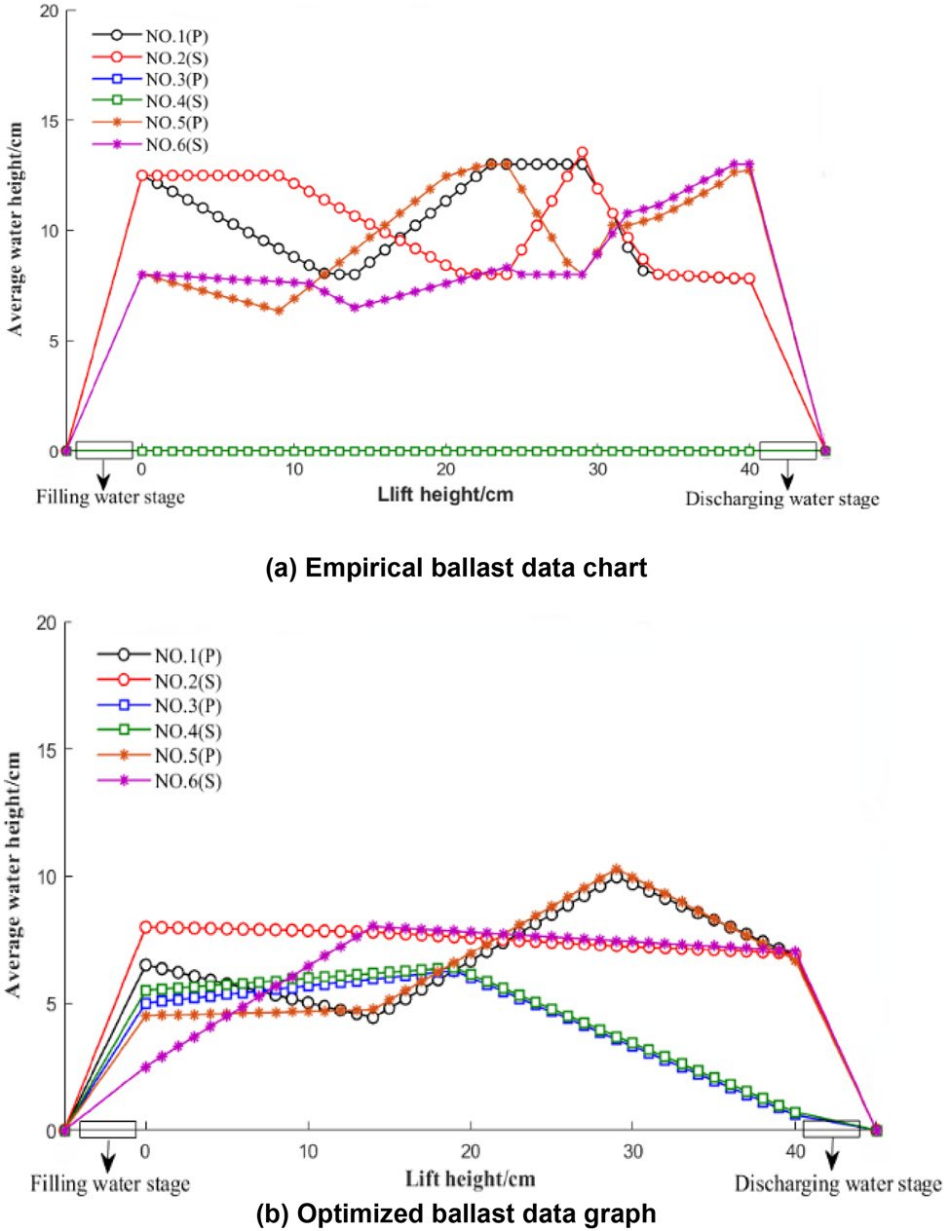
Change chart of the average water level of each ballast tank under experience and optimization schemes.

Figure 12a shows the ballast data map of the empirical average water level height change and Fig. 12b shows the optimized ballast data map of the average water level height change. This can be carried out by MATLAB programming. For the empirical ballast data, the initial preballast amount was 40 kg. The initial preballast amount for the optimized ballast data was 30 kg. During the preballasting process, the water level of the ballast water in the port and starboard ballast water tanks is adjusted to form a certain water level difference, resulting in a small-angle forward-leaning posture, which is convenient for the drowning person to climb the rescue net.

From Fig. 12a,b, it can be observed that the empirical scheme selects four ballast water tanks to participate in the deployment, and the optimization scheme mainly completes the deployment of ballast water through six ballast water tanks. For the optimal process shown in Fig. 12b, when the lift height ranges from 0 to 15, the average water level in the port 3 and 5 tanks increases, and the average water level in the port 1 tank decreases. The average water levels in cabins 4 and 6 on the starboard side increased, and the average water level in cabin No. 2 on the starboard side decreased. Because the experimental conditions in this range are that the drowning person first climbs the rescue net with their right hand, the gravity effect is simulated on the port side of the rescue net during the experiment. When the lifting height ranges from 15 to 30, cabins 1, 3, and 5 on the port side are at this time. The average water level also increases. The water levels in cabins 2, 4, and 6 on the starboard side decrease, and the average water level in cabin 4 on the starboard side first increases and then decreases. Because the hypothetical experimental conditions within this range are the process of the left and right hands and the body of the drowning person lying on the rescue net, to balance the floating state and stability of the rescue robot, when the lifting height ranges from 31 to 40, the port and starboard sides are the average water levels in cabins 1, 2, 3, 4, 5, and 6 all decrease to varying degrees. The hypothetical experimental condition in this range is to simulate the clockwise random additional force of the drowning person on the robot to increase the buoyancy to offset the force.

### Ballast time and quality comparison between experience and optimized solutions

**Figure 13:**
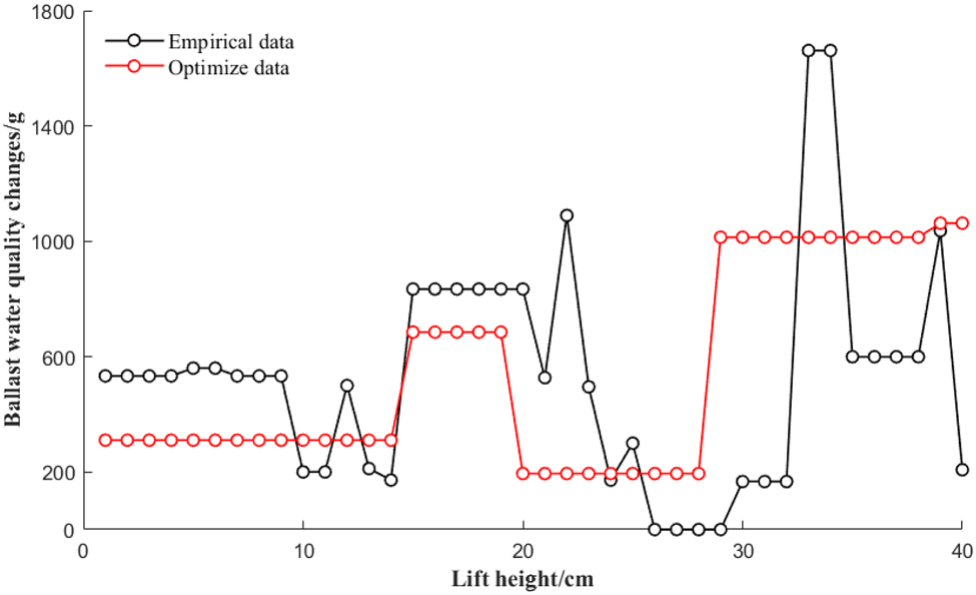
Comparison chart of the quality change of ballast water allocation.

**Figure 14:**
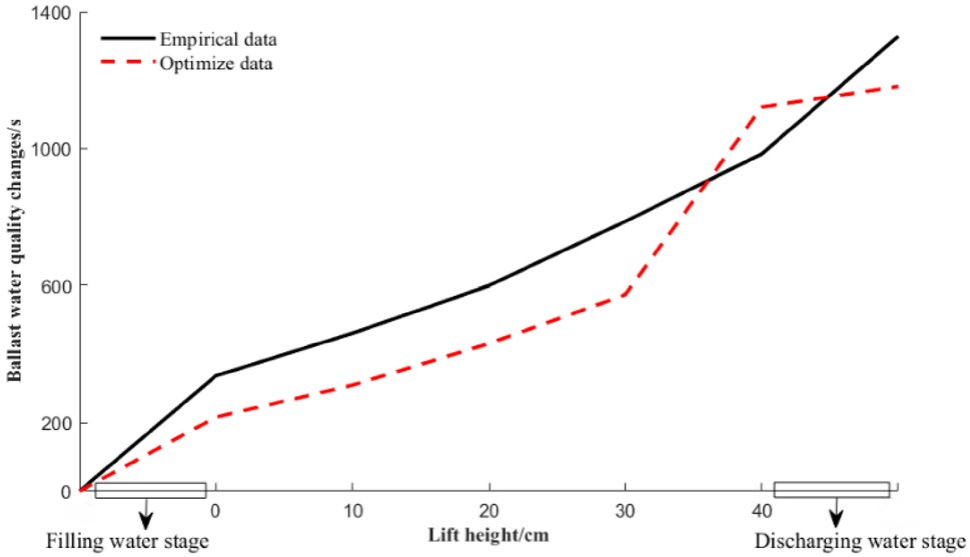
Ballast time change comparison chart.

Figure 13 shows a comparison of the changes in the total mass of the ballast water allocation based on experience and optimization schemes. The data show the relationship between the stage value of the lift height and the quality of the ballast water as the lift height increases. Figure 14 shows the change chart of the ballast time for each scheme after experience and optimization. From the comparison and analysis of Figs. 13 and 14, the total ballast water allocation amount of the empirical data and the optimized data are 37,204.3 g and 25,747.7 g, respectively, and the total ballast time of the empirical data and the optimized data are 1328 s and 1181 s, respectively. In summary, compared with the empirical data, the total ballast water allocation amount and total allocation time are reduced by 30.79% and 11.07%, respectively. The amount of ballast water tank deployment and utilization is greater than that of the empirical scheme, but it is better than the empirical scheme in terms of the total amount of ballast water and total deployment time.

### Influence of the initial ballast amount on time under the optimized scheme

**Figure 15:**
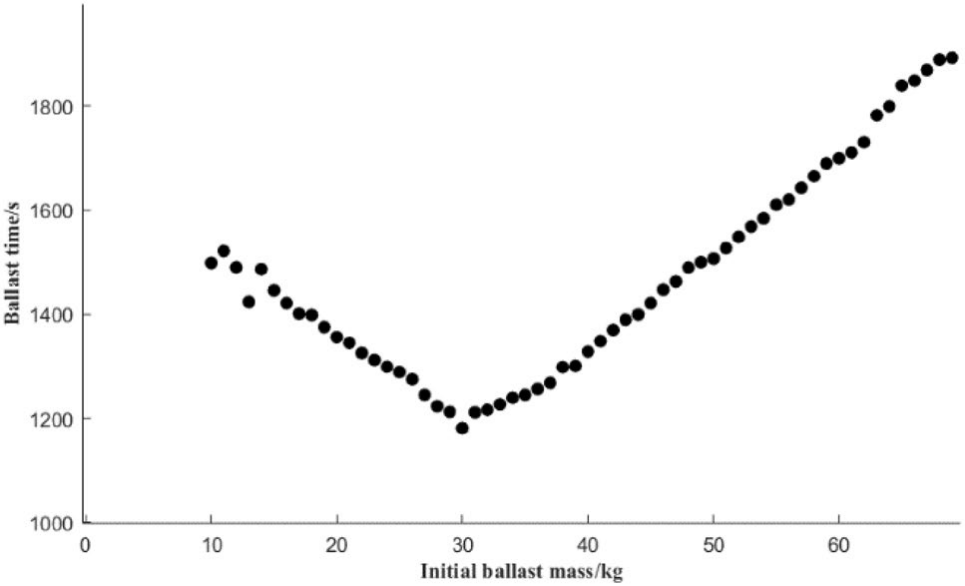
Influence of different initial ballast amounts on total deployment time.

The initial ballast amount refers to the mass of ballast water when the ballast water tank of the rescue robot is preballasted before rescue. For the whole process of salvage and ballast water allocation, in addition to maintaining the overall stability and balance after the salvage is successful, it is also necessary to adjust the attitude before salvage and salvage by adjusting the ballast water to break this equilibrium condition, and changing the stance in the water is adapted to the “salvage” rescue of the drowning person. Therefore, the size of the initial ballast also affects the preballast time *S*_1_ and deployment time *S*_*K*_ total deployment time. The effect of different initial ballast water ballast qualities on the total ballast time is shown in Fig. 15.

### Comparative analysis of the influence of experience and optimization schemes on the tilt angle

**Figure 16:**
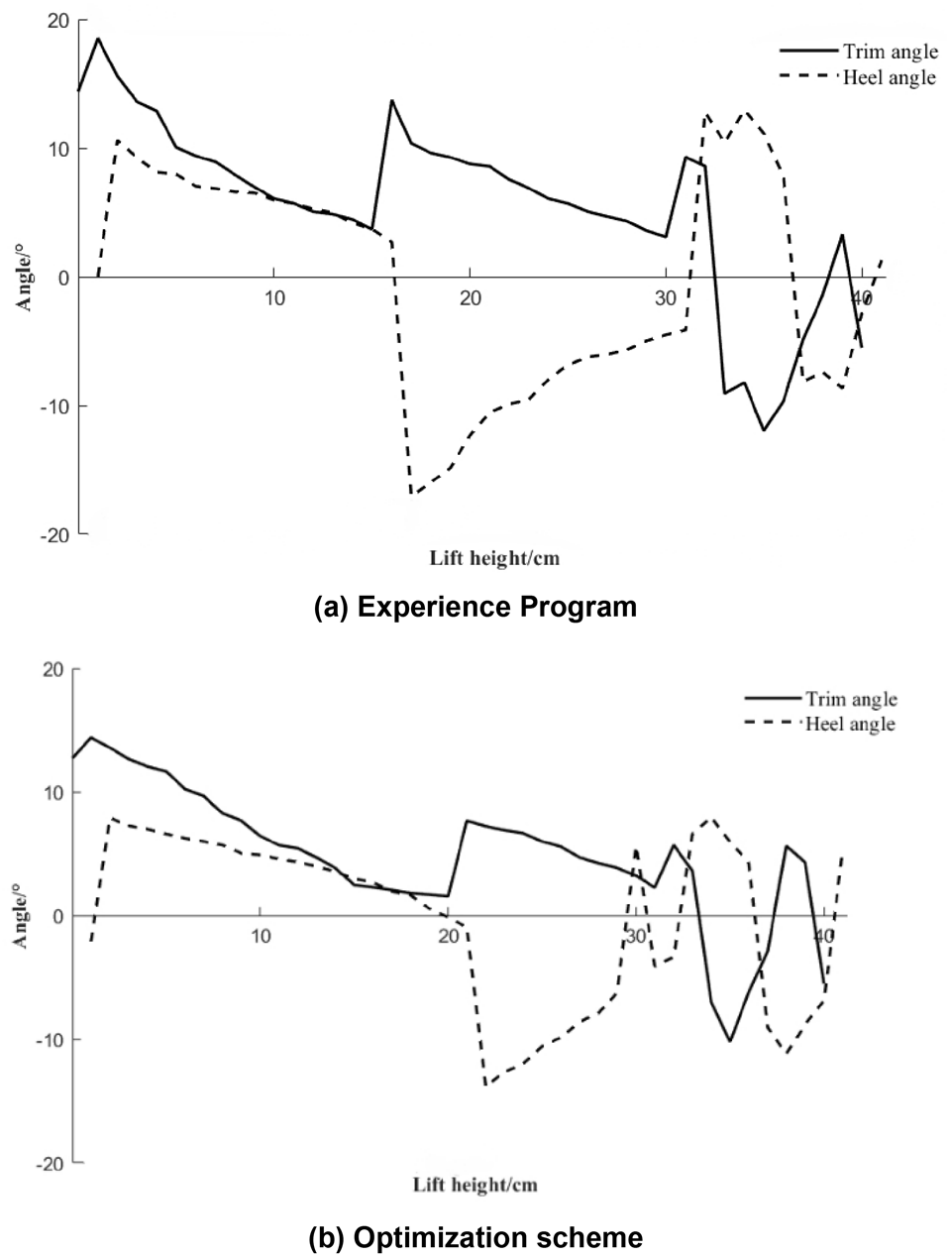
Comparison and analysis diagram of the heel and trim inclination change.

Figure 16a,b show comparative analysis diagrams of the changes in the heel and trim angles of the robot by the empirical and optimized schemes, respectively. It can be seen from Fig. 16 that the variation ranges of the heel and trim inclination angles under the empirical scheme and the optimized scheme are 17.05°, 15.8°, 12.83°, and 13.7°, which are reduced by 24.75% and 13.29%, respectively. The stability of the angle change was improved by 4.18% and 8.67% respectively. This indicates that, compared with the empirical scheme, the overall ballast deployment time, total ballast water deployment amount, and change trend of the inclination angle of the optimized scheme are relatively fast and stable.

### The impact of decision-making stage scope selection on deployment time

**Figure 17:**
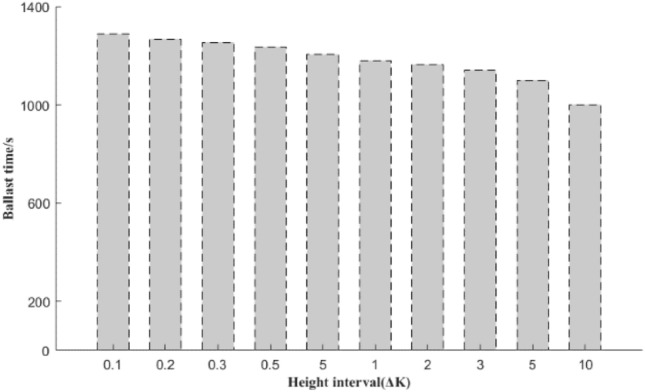
Comparison of different ∆*K* values.

When selecting the lift height interval range of the stage space *K*, the selection and change in the height interval affect the overall deployment time. Therefore, it is necessary to carry out a comparative analysis of this and discuss its impact on the overall deployment time under the range of each stage interval. This section divides the stage lift interval Δ*K* into 10, 5, 3, 2, 1, 0.5, 0.3, 0.2 and 0.1 to compare and analyse the optimal total deployment time in each state. The comparison results are shown in Fig. 17.

From the analysis in Fig. 17, the extreme difference time under different interval ranges is 279 s, which only accounts for 1.27% of the total allocation time under the optimal scheme. Therefore, the choice of the range of the different decision stages has less of an impact on the overall deployment time.

## Conclusion

1. To improve the ballast water allocation efficiency and stability of the attitude adaptive rescue robot designed in this paper in engineering practice, the lifting height of the robot in the whole rescue process is divided into a multistage decision-making problem, and a dynamic programming algorithm under the multistage background of dynamic decision-making. The optimization calculation method and optimization mathematical model of the rapid adjustment scheme of the wading rescue robot make up for the research gap in the related fields of the wading rescue robot. In the context of an example rescue, through MATLAB simulation and model robot experiments, the experience and optimization data in the context of virtual rescue are analysed. Compared with an empirical scheme, the total mixing time and the total mixing mass of ballast water in the optimization scheme are reduced by 11.07% and 30.79%, respectively, the change stability of heel and trim angles are improved by 4.18% and 8.67%, respectively, which verifies the effectiveness of the model method laying a foundation for the compilation, design and implementation of the follow-up computer automatic ballast program.
2. In the context of actual engineering rescue, because people are different from goods, they have the characteristics of strong random action. The simulated rescue dummy used in this study only summarizes a common drowning posture through relevant research, reports and examples and carries out numerical simulation and real-world model experiments. Moreover, in the simulation and model robot experiments, only the relevant theoretical formulas were deduced for the actual interference of external factors such as wind, current, and waves. Due to the experimental conditions and other reasons, the actual model robot experiments were insufficient. Therefore, future research should consider other rescue backgrounds and stability experiments under the disturbance of wind, current and waves. This will be further analysed as a future research direction.
3. The flow rate of the ballast water pump used in this study is only 2 L/min, which cannot meet the needs of practical engineering applications. Through experiments, it is verified that the change in ballast tank depth has little effect on the change in pump flow, so this error is ignored. Only the proposed optimization methods and mathematical models are analysed and compared. If the ballast water pump with suitable flow is used in engineering practice, the speed and efficiency of adjustment will be greatly improved, and the total deployment time will be shortened to less than 5 min, which is more in line with the actual needs of drowning rescue.

## Data Availability

The datasets generated and/or analysed during the current study are not publicly available due Involving related enterprises and government departments, but are available from the corresponding author on reasonable request.

## List of symbols

*K*: Stage space collection
*N*: Number of stages
*i*: Ballast tank number
*Z*: State space collection
*A*: Number of cycles
*D*: Action space collection
*V*: Speed
*λ*: Wavelength
*χ*: Wave direction angle
$\rho$: Density of ballast water
$$\rho_{air}$$: Air density
*g*: Gravitational acceleration
$\varphi$ and $\theta$: Heel angles and trim angles
$\alpha$ and ${\uppsi }$: Flow and yaw angles
G: Center of gravity of the robot
$x_{f}$: Height of the drift center on the water plane
*L*: Shape length
$L_{s}$: Total robot length
*B*: Profile width
Re: Reynolds number
$$\omega_{e}$$: Encounter frequency
$m_{W}$: Total mass of the loaded ballast water
$m_{p}$: Mass of the rescued person
$m_{i}^{P,S}$: Bottom area of the ith ballast tank on the port and starboard sides
*Z*_*N*_: State under the *N*th stage
$Z_{K}^{P,S}$: Collection of the average water level heights of each ballast tank at different stages
$d_{Kn}^{P,S}$: Average water level in the ith ballast tank on the port and starboard sides of the *N*th stage
$D_{Ki}^{P,S}$: Set of water level change actions of the ith ballast tank on the port and starboard sides of the *N*th stage
$d\left( x \right)$: Draught height at the cross section
$d_{i}^{P,S}$: Water level height of the ith water tank on the port and starboard sides
$d_{iH}^{P,S}$: Maximum height limit of the ith water tank on the port and starboard sides
$d_{\max }$, $d_{T\max }$, $d_{L\max }$ and $d_{S\max }$: Maximum draught limits of the robot in the upright float, heel, trim, and any inclination state
$d^{K}$, $d_{T}^{K}$, $d_{L}^{K}$ and $d_{S}^{K}$: Average draught of the robot in the upright float, heel, trim or any inclination state at stage *K*
$d_{{{\text{mi}} x}}$, $d_{{T{\text{mi}} x}}$, $d_{{L{\text{mi}} x}}$ and $d_{{S{\text{mi}} x}}$: Robot in upright float and heel, respectively, the maximum limit of draught in trim or any inclination
${\text{d}}_{FP}^{K}$, ${\text{d}}_{FS}^{K}$, ${\text{d}}_{SP}^{K}$ and ${\text{d}}_{SS}^{K}$: Port and starboard draughts on six sides
$s_{\max }^{K}$, $\theta_{\max }^{K}$ and $r_{\max }^{K}$: Robot’s maximum gyration speed, angle and radius
*ri*: Average water level growth
*lr*: Average water level decreases
*st*: Average level is flat
$D_{K}^{P,S}$: Set of decision-making effects of the *i-*th ballast tank on the port and starboard sides of the *N*-th stage under the action space *D*
$\Pi_{K}^{P,S}$: Variation of the average water level height in the strategic space
$\Delta d_{Ki}^{P,S}$: Average water level height changes of the ith ballast tank on the port and starboard sides of the *N*th stage
$F_{K}^{P,S}$: Total state transition function of the port and starboard sides in the Nth stage
$S_{K}^{P,S}$: Ballast time at stage *N*
$S_{w}$: Robot wet surface area
$Q_{1\max }^{P,S}$: Maximum limited ballast amount of preballast
$Q_{K}^{P,S}$: Total ballast time
$Q_{A}^{*P,S}$: Optimal collection of total ballast time under cycle A
$D_{K}^{*P,S}$: Optimal action strategy
$H_{{{\text{mix}}}}^{K}$ and $H_{{{\text{max}}}}^{K}$: Minimum and maximum lift heights
$\tau$: Inflow volume of the ship
$H^{K}$: Lift height at K-stage
$P_{\max }^{K}$: Maximum weight of the rescued person under the condition of no ballast
$P^{K}$: Weight of the rescued person
$\tau_{{\text{w}}}$, $\tau_{{\text{r}}}$, $\tau_{{\text{p}}}$ and $\tau_{{\text{h}}}$: Ballast moment of the ballast water, the tilt moment of the rescued person, the restoring moment of the robot and the disturbance moment of the external environment, respectively
$\tau_{{{\text{wx}}}}$, $\tau_{{{\text{wy}}}}$, $\tau_{{{\text{px}}}}$ and $\tau_{{{\text{py}}}}$: Component moments of the ballast water and rescued person the X- and Y-axis directions
$\tau_{wind}$, $\tau_{wave}$ and $\tau_{flow}$: Wind moment, wave moment and flow moment
$\Delta x_{i}^{p,s}$ and $\Delta y_{i}^{p,s}$: Moving distances of the centre of gravity of ballast water in the ith ballast tank on the port and starboard sides in the X- and Y-axis directions
$\Delta x_{p}$, $\Delta y_{p}$ and $\Delta z_{p}$: Moving distances of the rescued person centre of gravity in the X, Y and Z directions
$\Delta P$: Dynamic pressure caused by waves
$x_{w}$ and $y_{w}$: Transverse and transverse centre of gravity coordinates of the ballast water
$\overline{{G_{1} M}}_{NT}$ and $\overline{{G_{1} M}}_{NL}$: New transverse and longitudinal stability height after the rescue
$V_{{H_{L} }}$: Wind speed when the height of the horizontal plane is $H_{L}$
$$H_{Lr}$$: Reference height
$V_{{H_{Lr} }}$: Wind speed at the reference height
$${\Lambda }$$: Coefficient
$A_{\xi }$: Wind-receiving area of the $\xi$th module
$T_{s\xi }$ and $T_{h\xi }$: Shape and height factors of the $\xi$th module
$T_{D} \left( x \right)$: Drag coefficient of the robot cross-section at ordinate x
$T_{1}$: Model conversion coefficient
*T*: Shape factor
$T_{{\text{f}}}$: Friction resistance coefficient of the plate
$S_{L}$: Total number of divided modules
$i_{x}$: Lateral moment of inertia of the free surface area relative to its inclined axis
